# Spermatocytic Tumor Arising in a Cryptorchid Testis: A Case Report and Narrative Review

**DOI:** 10.3390/jcm15176797

**Published:** 2026-09-02

**Authors:** Laurențiu Augustus Barbu, Stelian-Stefaniță Mogoantă, Liliana Cercelaru, Marius Cristian Marinaș, Nicolae-Dragoș Mărgăritescu, Mihai Popescu, Valentina Căluianu, Gabriel Florin Răzvan Mogoș, Liviu Vasile, Tiberiu Stefăniță Țenea Cojan

**Affiliations:** 1 Department of Surgery, Railway Clinical Hospital Craiova, University of Medicine and Pharmacy of Craiova, 2 Petru Rares Street, 200349 Craiova, Romania; laurentiu.barbu@umfcv.ro (L.A.B.); valentina.andronache@yahoo.com (V.C.); gabriel.mogos@umfcv.ro (G.F.R.M.); tiberiu.tenea@umfcv.ro (T.S.Ț.C.); 2 Department of Surgery, Emergency County Hospital, University of Medicine and Pharmacy of Craiova, 2 Petru Rares Street, 200349 Craiova, Romania; ssmogo@yahoo.com (S.-S.M.); dmargaritescu@yahoo.com (N.-D.M.); vliviu777@yahoo.com (L.V.); 3 Department of Embryology and Anatomy, University of Medicine and Pharmacy of Craiova, 200349 Craiova, Romania; liliana.cercelaru@umfcv.ro; 4 Imaging Department, University of Medicine and Pharmacy, 200349 Craiova, Romania; mihai_popescu_rad@yahoo.com

**Keywords:** spermatocytic tumor, cryptorchidism, testicular germ cell tumor, magnetic resonance imaging, immunohistochemistry, classical seminoma

## Abstract

**Background:** Spermatocytic tumor (ST) is a rare non-GCNIS-derived testicular germ cell neoplasm that predominantly affects older men and generally follows an indolent course. Its occurrence in a cryptorchid testis is exceptionally uncommon. **Methods:** A narrative literature review was conducted using the PubMed database, including studies published up to August 2026. The search used combinations of the terms “spermatocytic tumor”, “spermatocytic seminoma”, “cryptorchidism”, “undescended testis”, “immunohistochemistry”, “molecular features”, “magnetic resonance imaging”, and “treatment”. **Results:** A 56-year-old man presented with a painless left inguinal mass corresponding to a cryptorchid testis. Serum tumor markers were normal. MRI demonstrated a well-circumscribed heterogeneous lesion with pseudocystic areas and enhancement of the solid component. Radical inguinal orchiectomy was performed. Histopathological examination revealed the characteristic triphasic cellular population without GCNIS, lymphovascular invasion, or sarcomatous transformation. Immunohistochemistry showed SALL4 and CD117 positivity and absence of OCT3/4 and D2-40 expression, supporting the diagnosis of ST and its distinction from classical seminoma. No recurrence or metastatic disease was detected during 12 months of follow-up. **Conclusions:** ST arising in a cryptorchid testis represents an exceptionally uncommon presentation. Integration of clinical, radiological, morphological, and immunohistochemical findings is essential for accurate diagnosis and distinction from classical seminoma, thereby avoiding unnecessary additional treatment.

## 1. Introduction

Testicular cancer accounts for approximately 1–1.5% of male malignancies and predominantly comprises germ cell tumors, which encompass a heterogeneous group of neoplasms with distinct biological and clinical characteristics. Although seminoma and non-seminomatous germ cell tumors represent the majority of cases, several uncommon entities are recognized, including spermatocytic tumor (ST), one of the rarest testicular germ cell neoplasms [1,2,3].

Spermatocytic tumor, formerly termed spermatocytic seminoma, has long been recognized as a biologically distinct germ cell neoplasm [2,4]. According to the current World Health Organization (WHO) classification, ST is classified as a non-GCNIS-derived (type III) germ cell tumor [5]. Its distinct cellular origin, immunophenotypic profile, and molecular alterations clearly distinguish it from classical seminoma and other GCNIS-derived germ cell tumors [2,5].

ST accounts for approximately 1% of testicular germ cell tumors and predominantly affects older men. It generally follows an indolent clinical course, with radical orchiectomy being curative in most patients; however, rare anaplastic or sarcomatous variants may exhibit aggressive behavior and metastatic potential [2,6].

Despite advances in its pathological and molecular characterization, evidence regarding ST remains limited because of its rarity and is largely derived from case reports and small retrospective series. Its occurrence in a cryptorchid testis is exceptionally uncommon, with only a very limited number of cases reported in the literature [2]. This unusual association is clinically relevant because cryptorchidism is a recognized risk factor for conventional testicular germ cell tumors, whereas its relationship with ST, a biologically distinct non-GCNIS-derived neoplasm, remains uncertain. We therefore report a rare case of ST arising in a cryptorchid testis, with integrated MRI–pathology correlation and immunohistochemical characterization, complemented by a focused review of its clinicopathological features and the potential significance of this unusual association.

## 2. Case Presentation

### 2.1. Patient Information

We report the case of a 56-year-old man with no significant medical history who was admitted to the Department of Surgery, Railway Clinical Hospital, Craiova, Romania, for evaluation of a progressively enlarging, painless left inguinal mass that had been present for approximately three months. The patient denied previous scrotal trauma, urinary symptoms, constitutional complaints, infertility, or any history of urological disorders.

The mass corresponded to a previously undescended left testis. No other relevant personal medical history was identified.

In accordance with the institutional guidelines of the Ethics Committee of the Railway Clinical Hospital Craiova, Romania, the study was reviewed and approved by the Ethics Committee (Approval No. 11213, 18 December 2025). Informed consent was obtained from the patient, whereby individual written informed consent was obtained, ensuring full compliance with ethical standards.

### 2.2. Clinical Findings

At admission, the patient was in good general condition, with stable vital signs and no cardiorespiratory abnormalities. Physical examination revealed a firm, painless mass in the left inguinal region, corresponding to the undescended left testis and measuring approximately 6 cm in diameter at the widest point.

The right testis was normally descended and showed no palpable abnormalities. No clinically detectable inguinal or supraclavicular lymphadenopathy was identified.

Routine laboratory investigations, including complete blood count, liver and renal function tests, inflammatory markers, and coagulation profile, were within normal limits. Serum tumor markers, including alpha-fetoprotein (AFP), beta-human chorionic gonadotropin (β-hCG), and lactate dehydrogenase (LDH), were within the reference ranges.

### 2.3. Timeline

The main events in the patient’s clinical course are summarized in Table 1, from the onset of symptoms to surgical treatment, pathological diagnosis, and subsequent follow-up.

### 2.4. Diagnostic Assessment

**Figure 1:**
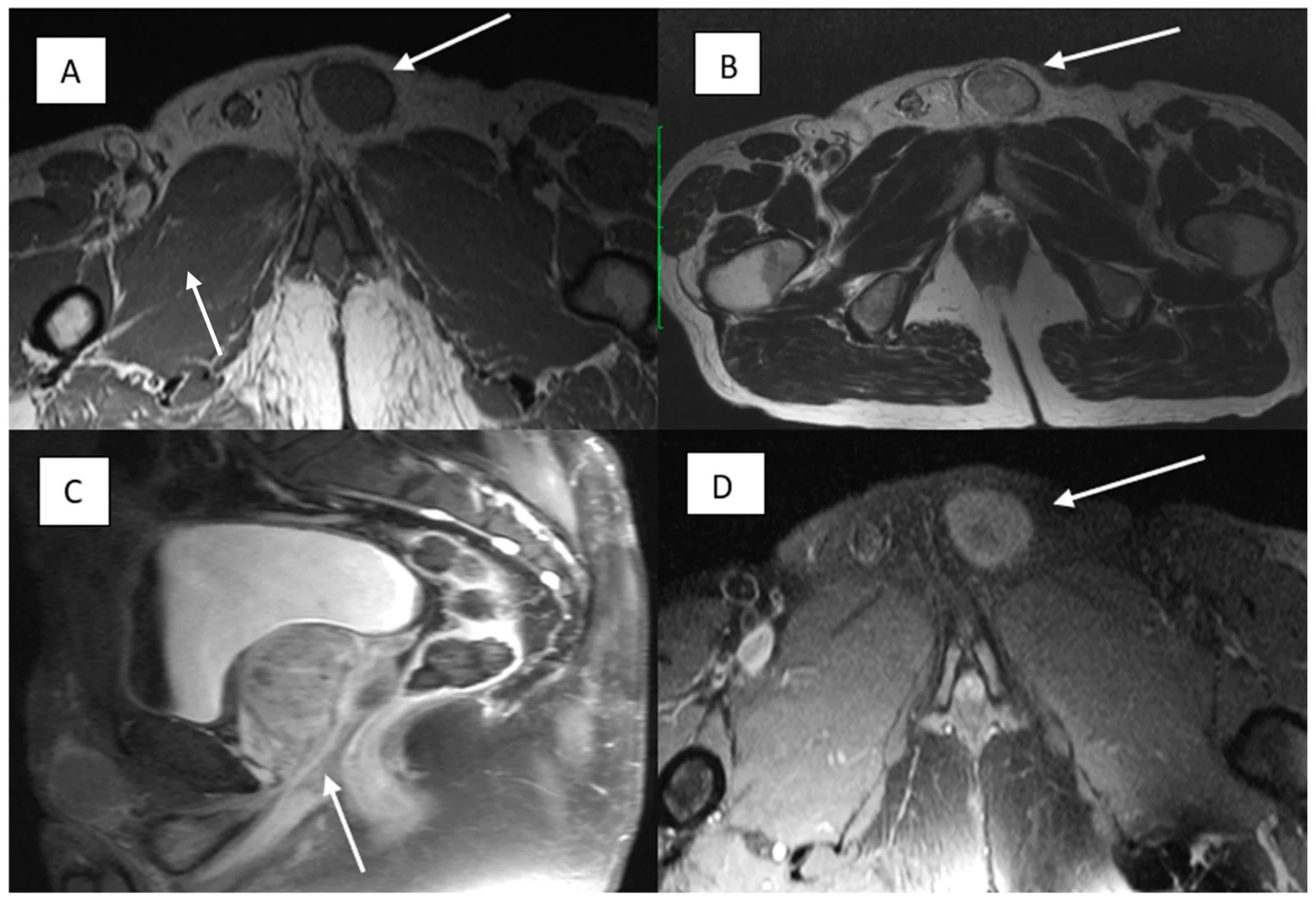
**Magnetic resonance imaging of the left cryptorchid testis. The white arrow identifies the left undescended (cryptorchid) testis.** (**A**,**B**) Native axial T1- and T2-weighted images demonstrating a relatively well-circumscribed intratesticular lesion with a heterogeneous pseudocystic appearance. (**C**,**D**) Post-contrast sagittal and axial T1-weighted images showing enhancement of the solid component, whereas the cystic component shows no contrast enhancement.

Scrotal ultrasonography demonstrated a heterogeneous solid lesion involving the left cryptorchid testis. To further characterize the lesion and evaluate its local extent, magnetic resonance imaging (MRI) was performed. Native axial T1- and T2-weighted sequences demonstrated a relatively well-defined lesion within the left cryptorchid testis, with a slightly heterogeneous, pseudocystic appearance. Following gadolinium administration, heterogeneous enhancement was observed within the solid component, whereas the cystic component showed no contrast uptake. No radiological evidence of invasion into the spermatic cord or adjacent soft tissues was identified (Figure 1A–D).

Contrast-enhanced computed tomography of the chest, abdomen, and pelvis demonstrated no retroperitoneal lymphadenopathy or distant metastatic disease.

Gross examination of the orchiectomy specimen revealed a well-circumscribed gray-white intratesticular tumor measuring 6.0 × 5.0 × 4.5 cm and replacing almost the entire testicular parenchyma. On the cut section, the lesion was homogeneous, soft, and gray-white, with focal pseudocystic degeneration and no macroscopic hemorrhage or necrosis.

**Figure 2:**
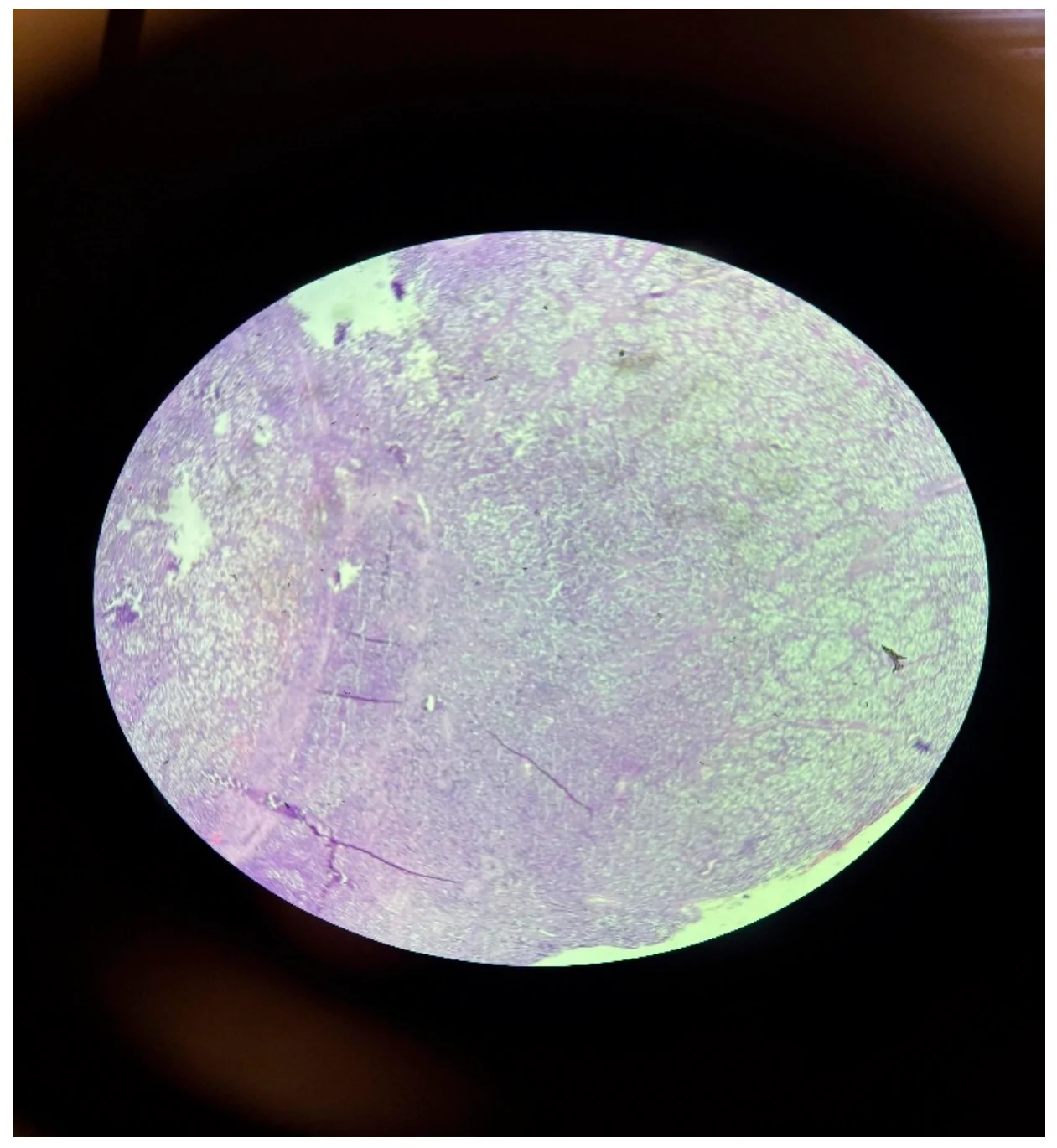
Low-power photomicrograph showing a well-circumscribed spermatocytic tumor composed of diffuse nodules separated by delicate fibrovascular septa. **H&E, ×40.**

**Figure 3:**
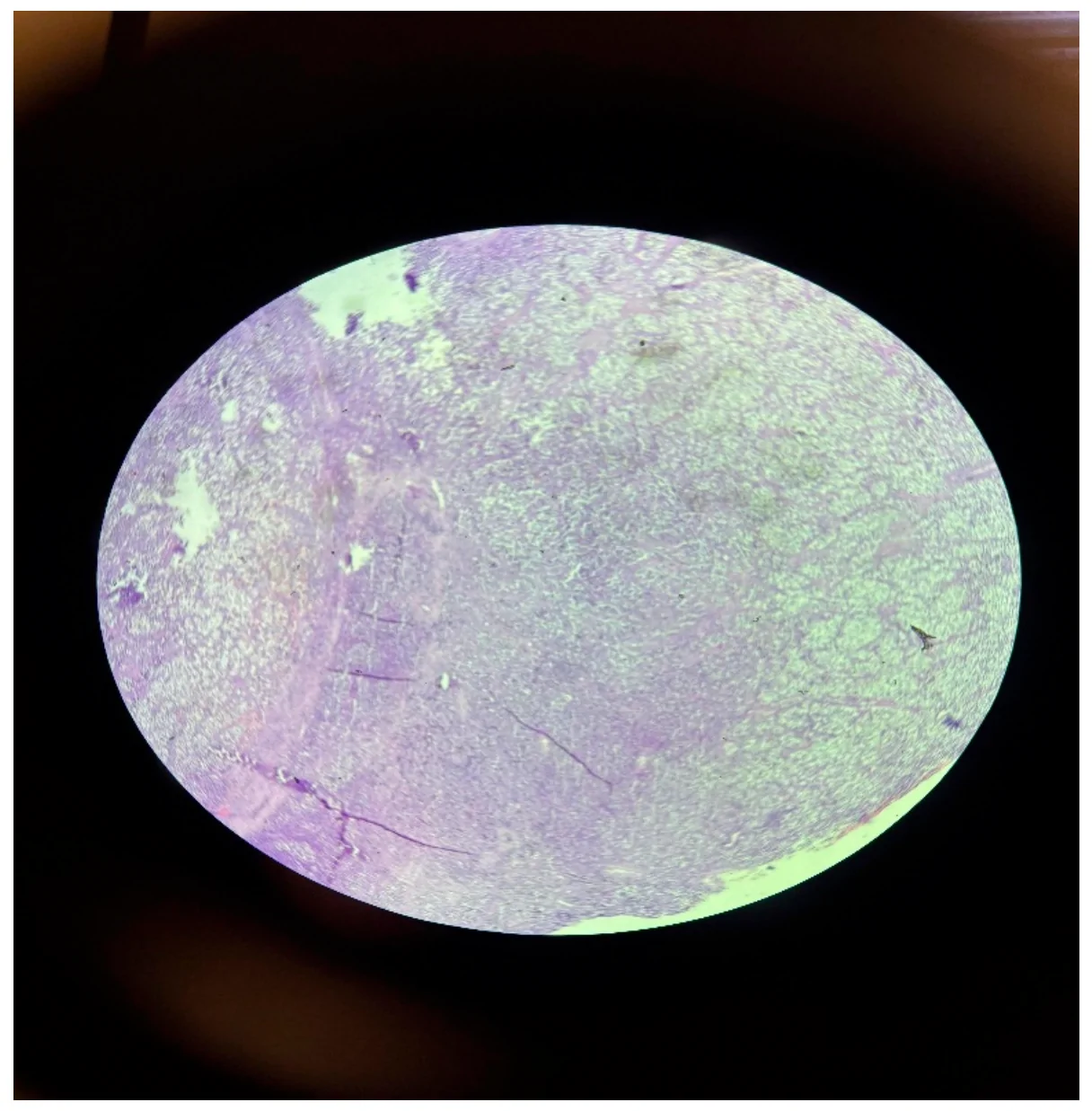
Intermediate-power photomicrograph illustrating the characteristic diffuse growth pattern of the neoplasm with thin fibrous septa. **H&E, ×100.**

**Figure 4:**
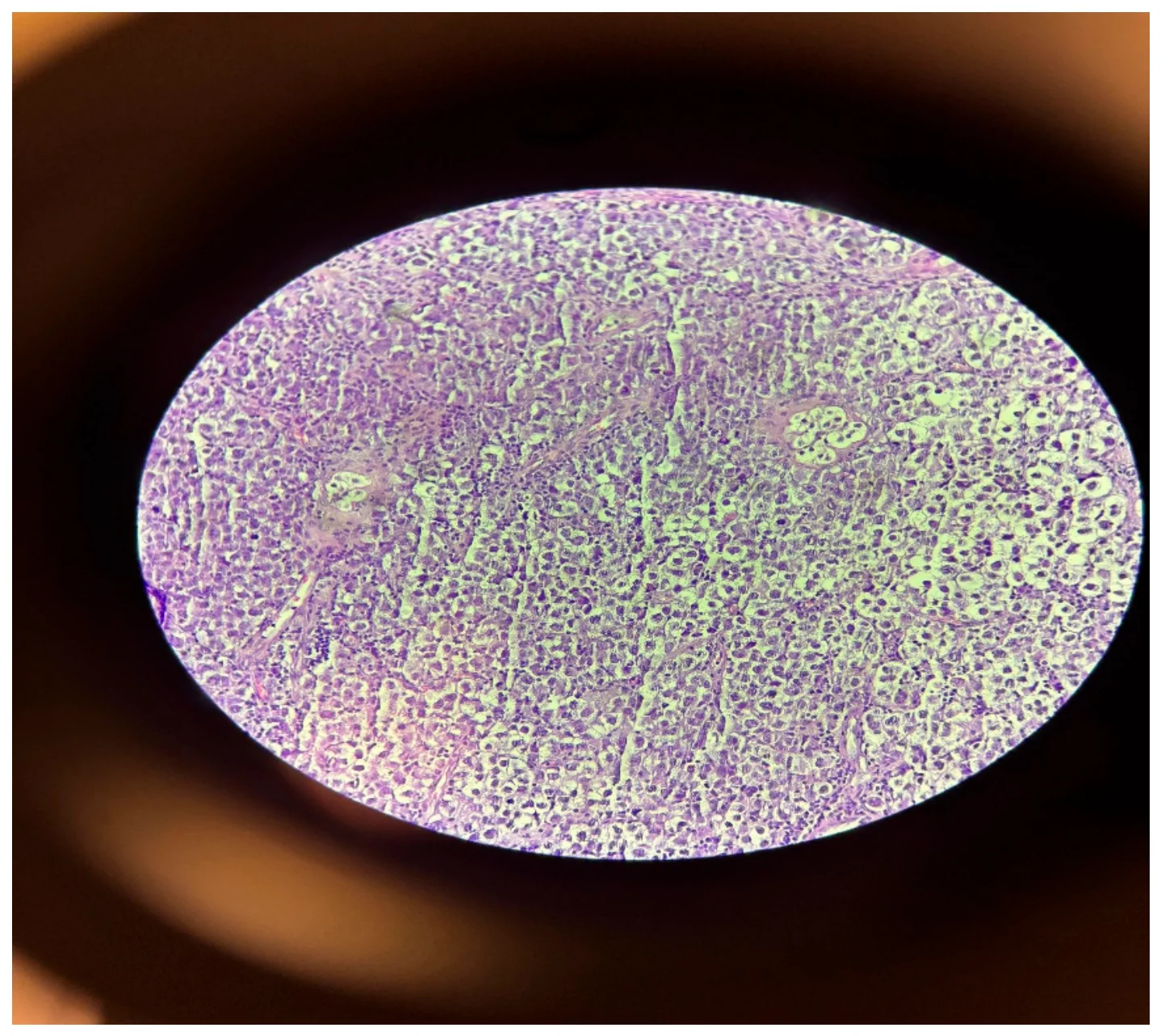
High-power photomicrograph demonstrating the characteristic triphasic cellular population of spermatocytic tumor, including small lymphocyte-like cells, predominant intermediate-sized cells with filamentous (“spireme-like”) chromatin, and scattered multinucleated giant cells. **H&E, ×400.**

Histopathological examination demonstrated a well-circumscribed neoplasm composed of diffuse sheets, cords, and nests of tumor cells separated by delicate fibrovascular septa containing scattered lymphocytes. Three characteristic cellular populations were identified: predominant intermediate-sized cells with clear to lightly eosinophilic cytoplasm, centrally located round nuclei, moderate anisokaryosis, conspicuous nucleoli, and finely filamentous (“spireme-like”) chromatin; small lymphocyte-like cells; and scattered large mononuclear and multinucleated cells. Occasional atypical mitotic figures were identified. No definite lymphovascular invasion, germ cell neoplasia in situ (GCNIS), extensive tumor necrosis, or sarcomatous transformation was observed (Figure 2, Figure 3 and Figure 4).

**Figure 5:**
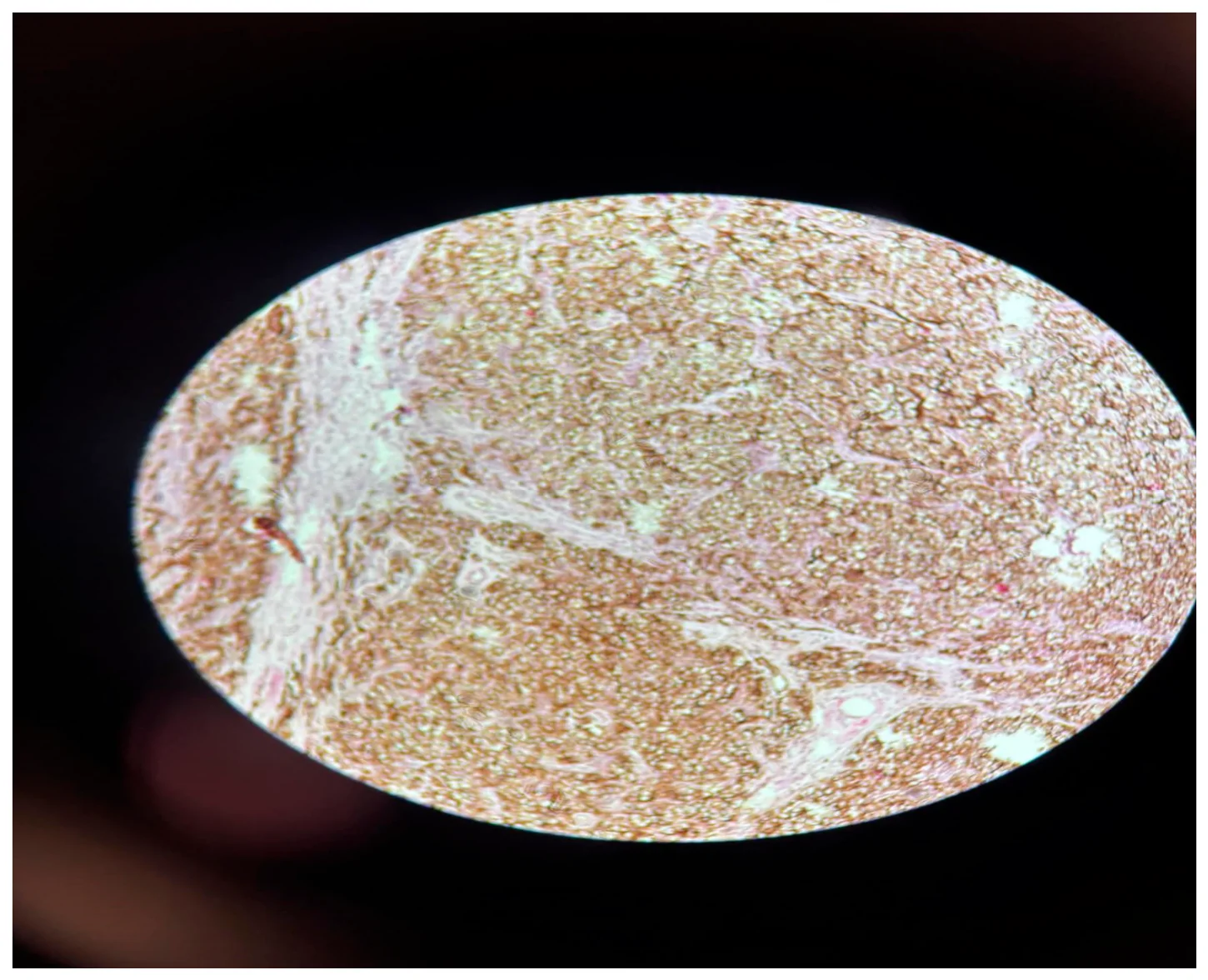
Diffuse nuclear immunoreactivity for **SALL4**, confirming the germ-cell origin of the neoplasm. Anti-SALL4 immunostaining, ×100.

**Figure 6:**
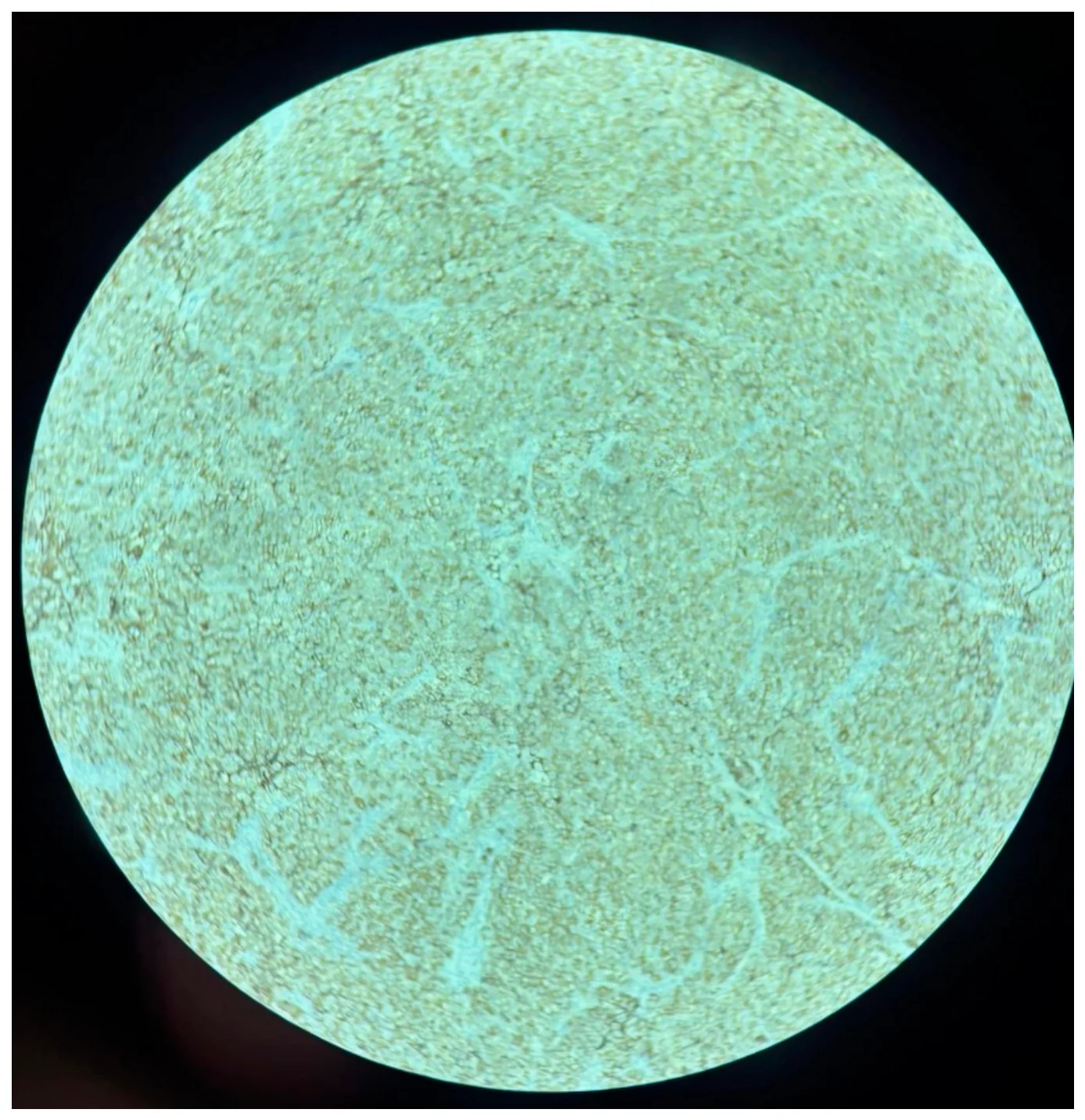
Diffuse membranous and cytoplasmic expression of **CD117 (KIT)** in the neoplastic cells. Anti-CD117 immunostaining, ×100.

**Figure 7:**
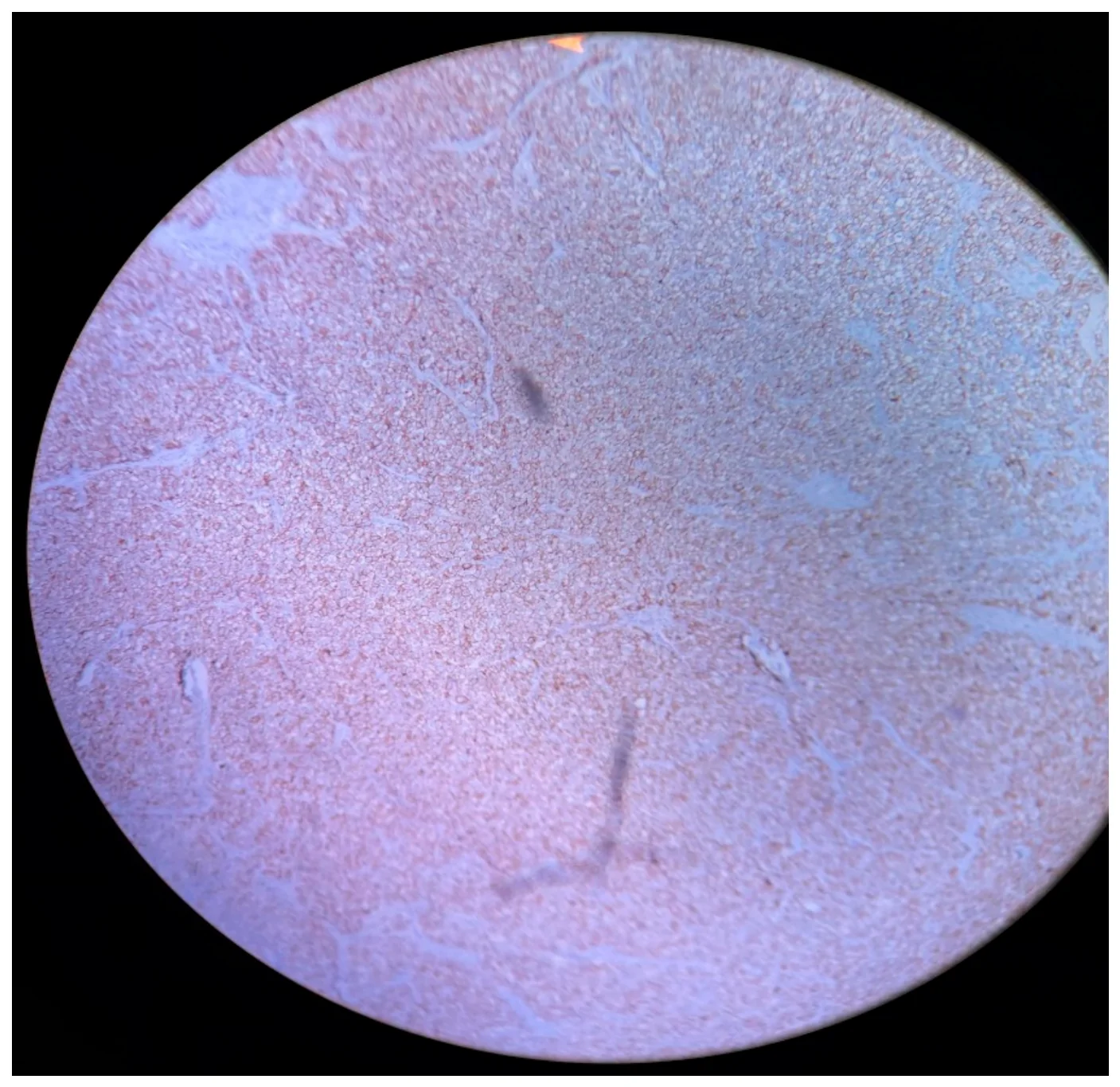
Negative nuclear immunostaining for OCT3/4 in the neoplastic cells, supporting the diagnosis of spermatocytic tumor and excluding classical seminoma. Anti-OCT3/4 immunostaining, ×100.

**Figure 8:**
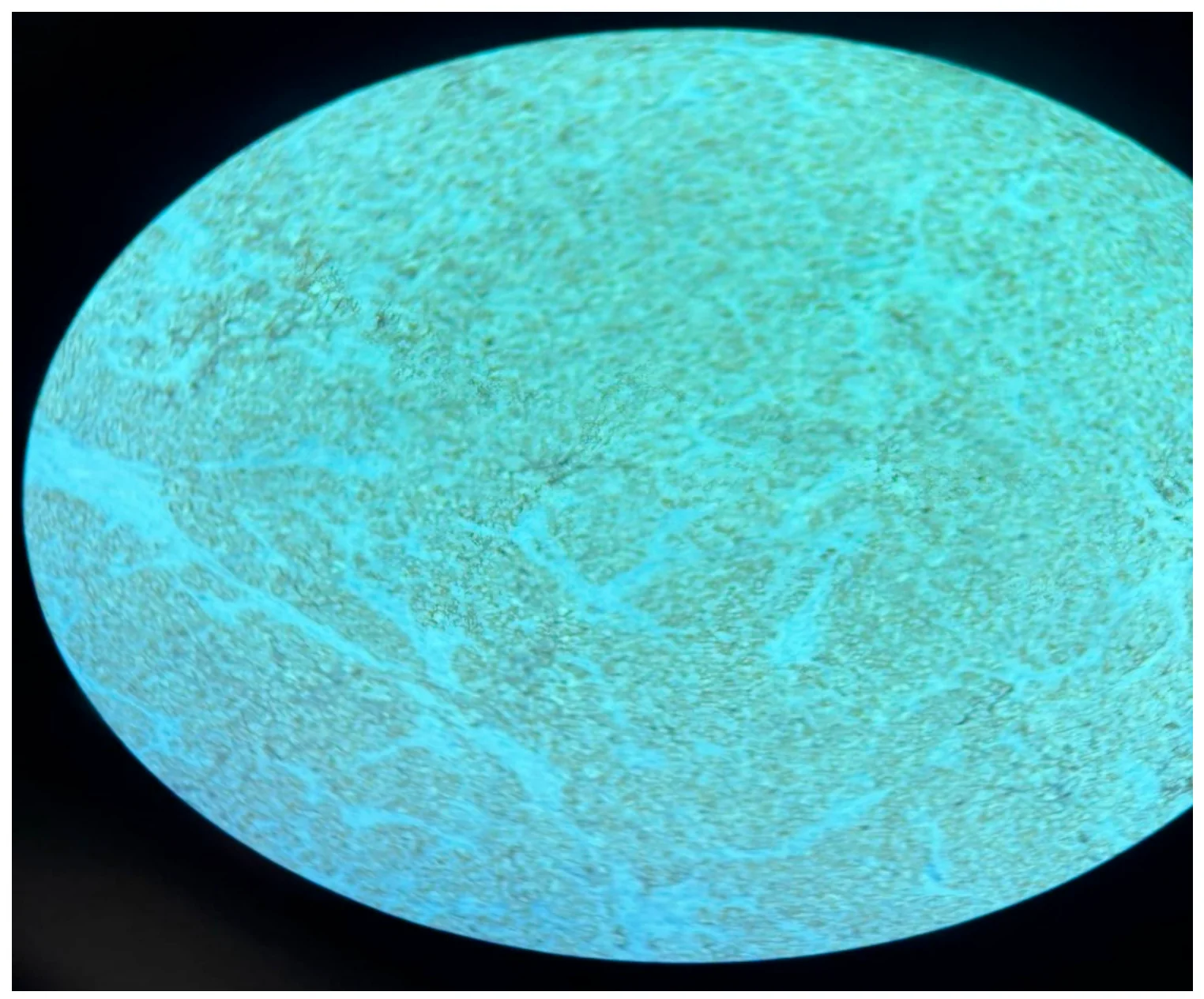
Negative **D2-40 (podoplanin)** immunostaining in the tumor cells, further supporting the diagnosis of spermatocytic tumor. Anti-D2-40 immunostaining, ×100.

Immunohistochemical analysis demonstrated diffuse nuclear expression of SALL4 and strong diffuse membranous and cytoplasmic positivity for CD117 (KIT). The neoplastic cells were negative for OCT3/4 and D2-40 (podoplanin) (Figure 5, Figure 6, Figure 7 and Figure 8). Integration of the morphological and immunohistochemical findings established the definitive diagnosis of spermatocytic tumor.

The main differential diagnosis was classical seminoma. The characteristic triphasic cellular population, absence of GCNIS, and immunophenotypic profile—particularly SALL4 and CD117 positivity combined with negative OCT3/4 and D2-40 expression—supported spermatocytic tumor rather than classical seminoma.

### 2.5. Therapeutic Intervention

**Figure 9:**
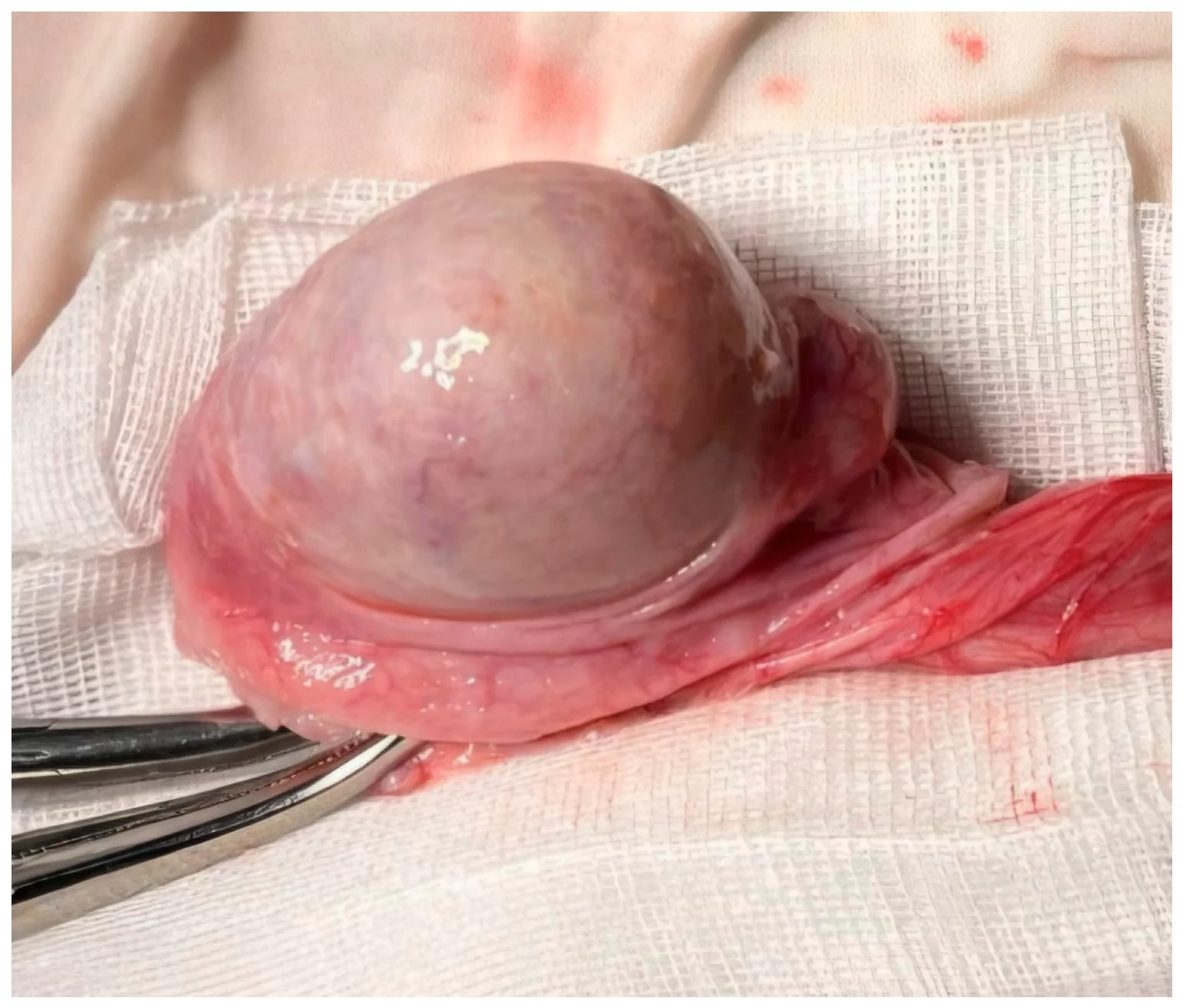
Intraoperative appearance of the left cryptorchid testis harboring an intratesticular tumor.

Based on the clinical and imaging findings, the patient underwent left radical inguinal orchiectomy. Intraoperatively, a markedly enlarged cryptorchid left testis measuring approximately 6 cm was identified. The external surface was smooth and intact, without macroscopic evidence of tunica albuginea rupture or extension into the spermatic cord or surrounding soft tissues (Figure 9).

Radical orchiectomy was performed en bloc according to standard oncological principles. No additional adjuvant oncological treatment was administered following the definitive pathological diagnosis.

### 2.6. Follow-Up and Outcomes

The postoperative course was uneventful, and the patient was discharged on the third postoperative day.

Following histopathological confirmation of spermatocytic tumor and in the absence of metastatic disease, lymphovascular invasion, GCNIS, or sarcomatous transformation, the patient was enrolled in a surveillance protocol consisting of periodic clinical examinations and imaging studies.

At the 12-month follow-up, no evidence of local recurrence or distant metastatic disease was detected.

### 2.7. Informed Consent

Written informed consent for publication of the clinical data and accompanying images was obtained from the patient.

## 3. Narrative Review

### 3.1. Incidence

Spermatocytic tumor (ST) is a rare testicular germ cell neoplasm, accounting for approximately 1% of testicular germ cell tumors and representing a distinct clinicopathological entity with characteristic epidemiological, molecular, and biological features [2,7]. Its rarity is supported by population-based data: an Australian registry of 9658 primary testicular tumors identified only 58 cases, corresponding to an age-standardized incidence of approximately 0.4 cases per million men per year [8,9].

### 3.2. Age Distribution

In contrast to classical seminoma, which predominantly affects younger adults, spermatocytic tumor occurs mainly in older men, with a reported median age at diagnosis of approximately 52–56 years. Cases in children and young adults are exceptionally rare [10]. Contemporary reports confirm a predominance during the fifth to seventh decades of life [11,12]. This distinctive age distribution represents a useful clinical feature in differentiating spermatocytic tumor from classical seminoma, which typically occurs at a younger age [1].

### 3.3. Risk Factors

The etiological factors underlying spermatocytic tumor remain poorly defined. Unlike conventional GCNIS-derived germ cell tumors, established risk factors such as cryptorchidism, infertility, and gonadal dysgenesis have not been consistently associated with spermatocytic tumor [3,13]. Although previous testicular cancer, cryptorchidism, family history, and infertility are recognized risk factors for testicular germ cell tumors in general, their relevance to spermatocytic tumor remains uncertain, supporting its distinct biological pathogenesis [14,15].

### 3.4. Molecular Pathogenesis

According to the current WHO classification, spermatocytic tumor is categorized as a non-GCNIS-derived (type III) germ cell tumor, reflecting its distinct developmental and molecular background [5]. Unlike GCNIS-derived germ cell tumors, ST lacks GCNIS and chromosome 12p gain or i(12p), frequently exhibits chromosome 9 alterations involving DMRT1, and shows differentiation toward primary spermatocytes rather than primordial germ cells or gonocytes [16,17,18]. Consistent with this classification, the ISUP molecular pathology consensus recognizes ST as a biologically distinct type III germ cell tumor, separate from prepubertal (type I) and GCNIS-derived (type II) tumors [19]. Although conventional ST generally follows an indolent course, anaplastic morphology and particularly sarcomatous transformation are associated with more aggressive behavior and poorer outcomes [2].

Molecular evidence further supports an origin from more differentiated male germ cells rather than primordial germ cells or gonocytes [20]. Gene-expression studies have demonstrated meiosis-associated markers, including TCFL5, CLGN, and LDHC, consistent with differentiation toward primary spermatocytes [21]. One of the most characteristic genomic abnormalities is chromosome 9 gain involving the DMRT1 locus, a key regulator of male germ-cell differentiation and meiosis [22].

Recurrent activating alterations involving HRAS and FGFR3 have also been identified and may contribute to tumorigenesis through signaling pathways involved in germ-cell proliferation [23]. The absence of GCNIS in the adjacent testicular parenchyma further supports the non-GCNIS-derived origin of ST [13], while the lack of chromosome 12p abnormalities, including i(12p), distinguishes it molecularly from postpubertal GCNIS-derived germ cell tumors [24].

Collectively, these cellular and molecular characteristics support the classification of spermatocytic tumor as a distinct non-GCNIS-derived testicular germ cell neoplasm.

### 3.5. Clinical Presentation

#### 3.5.1. Symptoms

Spermatocytic tumor typically follows an indolent course and most commonly presents as a slowly enlarging, painless unilateral testicular mass. The prolonged and often asymptomatic evolution may result in relatively large tumors at diagnosis; in a systematic review of 146 patients, the median tumor size was approximately 70 mm, with presentation predominantly related to local testicular symptoms [25]. Bilateral involvement is uncommon and, when present, is usually metachronous [1,7].

Pain, acute inflammatory manifestations, and systemic symptoms are uncommon in conventional localized tumors. In contrast, anaplastic or sarcomatous variants may exhibit rapid progression and metastatic disease, involving the retroperitoneal lymph nodes and lungs in particular [1].

#### 3.5.2. Serum Tumor Markers

Serum tumor markers are generally unremarkable in conventional spermatocytic tumor. AFP and β-hCG are typically within normal limits, whereas LDH may occasionally be elevated, particularly in patients with a greater tumor burden, although this finding is nonspecific [25,26]. An isolated LDH elevation has also been documented in individual cases despite normal AFP and β-hCG levels [3]. Beyond conventional serum tumor markers, circulating microRNAs have emerged as promising biomarkers for the diagnosis, surveillance, and treatment stratification of testicular germ cell tumors. However, their clinical utility has been investigated predominantly in GCNIS-derived germ cell tumors, and their potential role in rare non-GCNIS-derived neoplasms such as spermatocytic tumor remains to be established [27].

#### 3.5.3. Clinical Examination

Physical examination typically reveals a firm, enlarged, non-tender testicular mass, with an unremarkable contralateral testis and no clinically detectable lymphadenopathy in localized disease [3]. Hydrocele or scrotal enlargement may occasionally occur but is nonspecific. As the clinical presentation cannot reliably distinguish spermatocytic tumor from other testicular neoplasms, definitive diagnosis relies on the integration of imaging and histopathological evaluation.

### 3.6. Imaging Findings

#### 3.6.1. Ultrasonography

Scrotal ultrasonography is the first-line imaging modality for the evaluation of testicular masses. Spermatocytic tumors are generally described as well-defined, heterogeneous intratesticular lesions, occasionally containing cystic or degenerative areas. Although this appearance may differ from the more homogeneous hypoechoic pattern of classical seminoma, sonographic findings are nonspecific and cannot establish the diagnosis [1].

#### 3.6.2. Contrast-Enhanced Ultrasonography

Contrast-enhanced ultrasonography (CEUS) has emerged as a complementary imaging technique for the characterization of testicular masses by providing real-time assessment of lesion vascularity and enhancement patterns. A systematic review and meta-analysis including 419 patients with 428 testicular masses reported an accuracy of 0.96 for CEUS in differentiating neoplastic from non-neoplastic lesions, with a positive predictive value of 0.85. The reported accuracy for distinguishing malignant from benign lesions was also 0.96, with a negative predictive value of 0.91 [28]. Although CEUS findings specifically associated with spermatocytic tumor have not been established, this technique may provide useful complementary information when conventional ultrasonography is inconclusive. Nevertheless, definitive diagnosis of ST remains dependent on histopathological and immunohistochemical evaluation.

#### 3.6.3. Computed Tomography

Computed tomography is primarily used for staging rather than characterization of the primary tumor. CT of the chest, abdomen, and pelvis is particularly relevant for detecting nodal or distant metastases, which are uncommon in conventional spermatocytic tumors but may occur in aggressive variants, especially those with anaplastic or sarcomatous transformation [25]. Metastatic involvement has been reported in the retroperitoneal and mediastinal lymph nodes and lungs, including multiple pulmonary and mediastinal lesions in aggressive disease [12]. Given that metastatic recurrences are more likely to occur during the first years after diagnosis, CT may also have a role in the surveillance of selected high-risk patients [25,26].

#### 3.6.4. Magnetic Resonance Imaging

MRI is not routinely required when ultrasonography demonstrates a solid intratesticular mass but may serve as a problem-solving modality when sonographic findings are equivocal. It can assist in confirming the intratesticular origin of a lesion, defining local extent, and evaluating adjacent structures. However, MRI findings specifically reported for spermatocytic tumor remain limited, and no characteristic imaging pattern has been established. Consequently, MRI cannot reliably distinguish spermatocytic tumor from seminoma, lymphoma, or other solid testicular neoplasms [1].

#### 3.6.5. Positron Emission Tomography

Evidence supporting the use of ^18F-FDG PET/CT in spermatocytic tumor is limited, and it has no established role in the routine initial evaluation of localized disease. Its potential application may be restricted to selected patients with suspected metastatic or recurrent disease when conventional imaging is inconclusive. Given the scarcity of available data, its diagnostic and prognostic value in spermatocytic tumor remains undefined [29].

### 3.7. Histopathological and Immunohistochemical Features

#### 3.7.1. Macroscopic Features

Spermatocytic tumors are typically well circumscribed and confined to the testicular parenchyma. On sectioning, they are usually soft, friable, and whitish to yellow-tan, occasionally with multinodular, gelatinous, or small cystic areas. Necrosis and hemorrhage are uncommon in conventional tumors but may occur in large lesions or in tumors showing anaplastic or sarcomatous transformation [1,11]. The tumor may replace a substantial portion of the testicular parenchyma and can occasionally be multifocal [19,30].

Sarcomatous transformation produces a more heterogeneous appearance, with fleshy, hemorrhagic, or necrotic areas. Extensive sampling is therefore essential, particularly in large or heterogeneous tumors, to identify a potentially limited conventional spermatocytic component [31].

#### 3.7.2. Microscopic Features

The defining histological feature of spermatocytic tumor is a characteristic triphasic cellular population composed of small lymphocyte-like cells, predominant intermediate-sized cells, and scattered large or multinucleated giant cells. The intermediate cells typically display round nuclei with finely granular or filamentous “spireme-like” chromatin resembling primary spermatocytes. Tumor cells usually grow in diffuse sheets, solid nests, or multinodular arrangements within an edematous or myxoid stroma [1,2].

Unlike classical seminoma, spermatocytic tumor generally lacks prominent fibrovascular septa, dense lymphocytic infiltration, granulomatous inflammation, and clear glycogen-rich cytoplasm. GCNIS is absent, although intratubular extension may occur and should not be misinterpreted as GCNIS [7,19,30].

Anaplastic tumors show increased cellularity, nuclear atypia, prominent nucleoli, and increased mitotic activity while retaining at least focal features of conventional spermatocytic tumor. Sarcomatous transformation is characterized by an abrupt transition to a high-grade sarcomatous component and is clinically important because of its association with metastatic behavior and poor prognosis [2,11,32].

#### 3.7.3. Immunohistochemical Features

The immunophenotype of spermatocytic tumor reflects differentiation toward spermatogonia and early spermatocytes. SALL4 is generally strongly expressed, supporting germ-cell differentiation, whereas CD117/c-KIT shows variable positivity and should not be interpreted in isolation because it is also commonly expressed in classical seminoma [2,33].

Spermatocytic tumors are typically negative for OCT3/4, PLAP, D2-40/podoplanin, CD30, AFP, glypican-3, β-hCG, and broad-spectrum cytokeratins [13]. The combination of SALL4 positivity with absent OCT3/4, PLAP, and D2-40 expression is particularly useful in distinguishing spermatocytic tumor from classical seminoma [2,33]. Additional markers associated with spermatogonial or spermatocytic differentiation include MAGE-A4, OCT2, SSX, SAGE1, SYCP1, XPA, DMRT1, FGFR3, and HRAS, although these are not routinely required for diagnosis [2,34,35].

The main immunohistochemical differences between spermatocytic tumor and classical seminoma are summarized in Table 2.

In tumors with sarcomatous transformation, focal SALL4 or KIT expression may be retained. Rhabdomyosarcomatous differentiation can be demonstrated by expression of desmin, myogenin, and MYOD1 [7].

#### 3.7.4. Molecular Features

Molecular studies further support the distinction between spermatocytic tumor and GCNIS-derived germ-cell tumors. Gain of chromosome 9 represents the most characteristic chromosomal abnormality and is associated with increased dosage and expression of DMRT1, a transcription factor involved in male germ-cell differentiation [36].

In contrast to classical seminoma and other postpubertal GCNIS-derived germ-cell tumors, spermatocytic tumor lacks chromosome 12p gain, including isochromosome 12p [i(12p)], consistent with a distinct pathway of tumor development [1,13,20]. Recurrent activating alterations involving FGFR3 and HRAS have also been described and may be related to “selfish spermatogonial selection,” providing a potential biological explanation for the predominance of these tumors in older men [23]. Overall, chromosome 9 gain, DMRT1 dysregulation, recurrent FGFR3/HRAS alterations, absence of GCNIS, and lack of 12p abnormalities support classification of spermatocytic tumor as a distinct non-GCNIS-derived germ-cell neoplasm [37].

#### 3.7.5. Differential Diagnosis

The principal differential diagnosis is classical seminoma, which can be distinguished by its association with GCNIS and expression of OCT3/4, PLAP, and D2-40 [1,19]. Other considerations include embryonal carcinoma, testicular lymphoma, sex cord–stromal tumors, and metastatic neoplasms. Embryonal carcinoma typically shows greater cytological atypia and expression of CD30 and OCT3/4 [38,39,40], whereas lymphoma and sex cord–stromal tumors are distinguished by their characteristic morphology and lineage-specific immunophenotype. Metastatic tumors require correlation with clinical history and appropriate immunohistochemical findings [41].

### 3.8. Treatment and Prognosis

Radical inguinal orchiectomy is the standard treatment for localized spermatocytic tumor and is curative in the vast majority of patients. Because most tumors present as stage I disease and follow an indolent course, routine adjuvant therapy is not indicated [2,42]. Evidence supporting testis-sparing surgery is currently insufficient [42].

Systemic treatment has a limited role in advanced disease. Metastatic tumors, particularly those with sarcomatous transformation, generally show limited responsiveness to conventional cisplatin-based chemotherapy, although occasional responses have been reported [2,20]. Similarly, current evidence does not support routine radiotherapy for either localized or metastatic spermatocytic tumor [43,44,45].

The prognosis of conventional spermatocytic tumor is excellent, with metastatic spread being exceptionally rare. Follow-up generally relies on clinical assessment and imaging, with closer surveillance warranted in patients with adverse pathological features, including sarcomatous transformation, anaplastic morphology, or large primary tumors [44,46]. In contrast to conventional tumors, anaplastic tumors and particularly those with sarcomatous transformation may exhibit aggressive behavior, treatment resistance, and substantially poorer survival [2,7,25].

## 4. Discussion

Spermatocytic tumor is a rare and biologically distinct testicular germ cell neoplasm that predominantly affects older men and generally follows an indolent clinical course [2,3,8,9]. Available evidence remains limited because of its rarity and is derived largely from case reports and relatively small retrospective series [2,3,9,31]. Against this background, the principal feature distinguishing the present case is the occurrence of ST in a cryptorchid testis, together with detailed radiological, morphological, and immunohistochemical characterization.

Cryptorchidism is a well-established risk factor for conventional testicular germ cell tumors, particularly seminoma [15,47], but its relationship with ST remains uncertain. A comprehensive review published in 2023 identified only two previously reported cases of ST arising in cryptorchid testes [2], indicating that this association is exceptionally uncommon. This rarity is particularly noteworthy because ST differs biologically from conventional postpubertal germ cell tumors: it is not associated with GCNIS and follows a distinct developmental and molecular pathway [4,5,19,35]. Therefore, mechanisms linking cryptorchidism to conventional GCNIS-derived tumors cannot necessarily be extrapolated to ST.

Nevertheless, the occurrence of ST in an undescended testis raises the possibility that the altered testicular microenvironment associated with cryptorchidism could influence neoplastic transformation beyond the conventional GCNIS pathway. Abnormal testicular position is associated with prolonged exposure to an altered thermal environment and impaired spermatogenesis, factors that may affect germ-cell homeostasis. Given the proposed origin of ST from more differentiated spermatogonial/spermatocytic germ cells, it is conceivable that long-standing alterations in the cryptorchid testicular environment could interact with age-related or intrinsic molecular events involved in ST development. However, this interpretation remains hypothetical. The extreme rarity of reported ST in cryptorchid testes, despite the established association between cryptorchidism and testicular cancer in general, does not support considering cryptorchidism an established risk factor for ST. Additional well-characterized cases, ideally incorporating molecular analysis, are required to determine whether this association has pathogenetic significance or is merely coincidental.

Preoperative imaging represents another relevant aspect of the present case. MRI demonstrated a relatively well-circumscribed heterogeneous lesion with pseudocystic areas and enhancement of the solid component, without evidence of spermatic cord or adjacent soft-tissue invasion. MRI findings specifically reported for ST remain limited [1,2,12]; therefore, the radiological–pathological correlation in this case contributes additional descriptive information regarding this uncommon presentation. Nevertheless, these imaging findings are not specific, and definitive classification continues to depend on morphological and immunohistochemical evaluation [44,45].

In the present case, the characteristic triphasic cellular population, absence of GCNIS, and immunophenotypic profile supported the diagnosis of ST and its distinction from classical seminoma. SALL4 and CD117 positivity, together with absent OCT3/4 and D2-40 expression, provided the most relevant diagnostic evidence [2,9,29,32,33,35]. These findings were also important in avoiding potential diagnostic confusion with GCNIS-derived germ cell tumors and, consequently, unnecessary additional treatment. Molecular testing for characteristic alterations associated with ST, including chromosome 9/DMRT1 abnormalities, FGFR3 or HRAS alterations, and chromosome 12p gain or i(12p), was not performed in the present case. Thus, although the morphological and immunohistochemical findings were sufficient to establish the diagnosis, molecular characterization would have been particularly informative in exploring whether ST arising in a cryptorchid testis shares the molecular profile described in conventionally located tumors.

Radical inguinal orchiectomy was adequate treatment in the present patient, in keeping with the generally favorable behavior of localized conventional ST [2,25,41,43]. Importantly, no GCNIS, lymphovascular invasion, extensive necrosis, or sarcomatous transformation was identified. The absence of adverse pathological features was consistent with the subsequent clinical course, with no evidence of local recurrence or distant metastatic disease during the 12-month follow-up period [7,25].

The present report has several limitations. First, the 12-month follow-up period is relatively short and does not allow for definitive assessment of long-term oncological outcomes. Second, the narrative nature of the literature review, with the search restricted to PubMed, may have resulted in the omission of relevant reports indexed in other databases and does not provide the methodological rigor of a systematic review. In addition, molecular testing was not available in the present case, preventing direct investigation of whether the unusual cryptorchid location was associated with distinctive molecular alterations. Finally, as a single case, this report cannot establish a causal relationship between cryptorchidism and ST. Accordingly, the proposed biological association should be regarded as hypothesis-generating rather than evidence that cryptorchidism represents a risk factor for this tumor.

The principal contribution of this case lies in documenting ST in an exceptionally uncommon anatomical and biological setting. Together with the very limited number of previously reported cases [2], this observation raises the question of whether the altered microenvironment of the cryptorchid testis may occasionally contribute to tumorigenesis in germ-cell lineages outside the conventional GCNIS pathway. Further molecularly characterized cases are needed to determine whether this association reflects a pathogenetically meaningful relationship or a rare coincidental finding.

## 5. Conclusions

Spermatocytic tumor is a rare non-GCNIS-derived testicular germ cell neoplasm with distinct clinicopathological and immunophenotypic characteristics and a generally favorable prognosis following radical orchiectomy. The present case is noteworthy for its occurrence in a cryptorchid testis, an exceptionally uncommon presentation, and for the correlation of preoperative MRI findings with characteristic histopathological and immunohistochemical features. Accurate recognition is essential to distinguish spermatocytic tumor from classical seminoma and other testicular neoplasms and to avoid unnecessary additional treatment. This case adds to the limited literature on spermatocytic tumor and underscores the diagnostic value of integrating clinical, radiological, morphological, and immunohistochemical findings.

## Figures and Tables

**Table 1:** Timeline of the patient’s clinical course.

Time	Clinical Event
**Approximately 3 months**	Onset of a progressively enlarging, painless left inguinal mass
**Admission**	Clinical examination and laboratory investigations; serum AFP, β-hCG, and LDH within normal limits
**Preoperative assessment**	Ultrasonography demonstrated a heterogeneous solid lesion involving the left cryptorchid testis
**Preoperative assessment**	MRI demonstrated a well-circumscribed heterogeneous lesion with pseudocystic areas and enhancement of the solid component
**Preoperative staging**	Contrast-enhanced CT of the chest, abdomen, and pelvis showed no retroperitoneal lymphadenopathy or distant metastatic disease
**Day 0**	Left radical inguinal orchiectomy
**Postoperative day 3**	Uneventful recovery and hospital discharge
**Histopathological assessment**	Morphological and immunohistochemical findings confirmed spermatocytic tumor
**12 months**	No evidence of local recurrence or distant metastatic disease

Abbreviations: AFP, alpha-fetoprotein; β-hCG, beta-human chorionic gonadotropin; CT, computed tomography; LDH, lactate dehydrogenase; MRI, magnetic resonance imaging.

**Table 2:** Comparative Immunohistochemical Features of Spermatocytic Tumor and Classical Seminoma.

Marker	Spermatocytic Tumor	Classical Seminoma
**SALL4**	Positive	Positive
**CD117/c-KIT**	Variable/positive	Positive
**OCT3/4**	Negative	Positive
**PLAP**	Negative	Positive
**D2-40**	Negative	Positive
**DMRT1**	Positive	Usually negative
**FGFR3**	Positive	Negative
**HRAS**	Positive	Negative

## Data Availability

The data presented in this study are available on request from the corresponding author. The data are not publicly available due to patient confidentiality.
